# From diagnosis to refusal-causes and barriers to organ donation after brain death: a cross-sectional study

**DOI:** 10.1590/1516-3180.2025.3503.27032026

**Published:** 2026-08-21

**Authors:** João Paulo Victorino, Vagner Pires de Campos, Victor Nei Vasconcelos Monteiro, Karina Dal Sasso Mendes, Emanuele Seicenti de Brito, Carla Arena Ventura

**Affiliations:** I PhD. Critical Care Nurse Specialist, Einstein Hospital Israelita; Escola de Enfermagem, Universidade de São Paulo (USP), Ribeirão Preto (SP), Brazil.; II Specialist. Physioterapist, Einstein Hospital Israelita; PhD Student, Faculdade de Medicina, Universidade de São Paulo (FMUSP), São Paulo (SP), Brazil.; III Specialist. MA Student, Escola de Enfermagem, Universidade de São Paulo (USP), Ribeirão Preto (SP), Brazil.; IV PhD. Professor, Escola de Enfermagem, Universidade de São Paulo (USP), Ribeirão Preto (SP), Brazil.; V PhD. Postdoctoral Student, Escola de Medicina, Universidade de São Paulo (USP), Ribeirão Preto (SP), Brazil.; VI PhD. Professor, Escola de Engermagem, Universidade de São Paulo (USP), Ribeirão Preto (SP), Brazil.

**Keywords:** Brain Death, Organ Transplantation, Public Health, Tissue and Organ Procurement, Critical Care, Transplantation, Bioethics, Multiprofessional Team

## Abstract

**BACKGROUND:**

Brain death is the essential condition for organ procurement, yet donation rates in Brazil remain insufficient to meet national transplant needs. Understanding both the clinical determinants of brain death and the social or institutional barriers to organ donation is crucial for improving donation outcomes and informing public health strategies.

**OBJECTIVES:**

To identify the main causes of brain death and analyze the factors associated with organ non-donation at a university hospital in São Paulo State.

**DESIGN AND SETTING:**

This cross-sectional study with a quantitative approach was conducted at a university hospital in São Paulo State, using data from the Organ Procurement Organization affiliated with the Universidade de São Paulo.

**METHODS:**

The medical records of all patients diagnosed with brain death between 2001 and 2015 were reviewed. Sociodemographic variables, clinical causes of brain death, and factors related to donation outcomes were assessed using descriptive statistics and chi-square tests.

**RESULTS:**

In total, the data of 1,688 cases were analyzed, including those of 574 donors (34%) and 1,114 non-donors. Traumatic brain injury and hemorrhagic stroke were the predominant neurological causes of brain death. No significant association was observed between sex or cause of brain death and donation outcome. Public initiatives such as the 2001 consent law and the 2014 “Green September” campaign showed limited short-term impact on donation rates.

**CONCLUSION:**

Although neurological causes account for most cases of brain death, donation outcomes are strongly influenced by social and institutional factors-particularly family refusal and trust in the healthcare system.

## INTRODUCTION

The shortage of organs for transplantation constitutes one of the main challenges facing health systems worldwide, despite advances in public policies and donation and transplantation programs. The World Health Organization recognizes the chronic insufficiency of organs as a global problem, highlighting that the demand for transplants significantly exceeds supply in nearly all countries.^1^ In Brazil, organ donation depends, among other factors, on the recognition and reporting of brain death, the appropriate implementation of the diagnostic protocol, and family authorization for donation.^2^

Although the country has one of the largest public transplantation networks worldwide, with national coverage and regulation under the Unified Health System, Brazil still has a considerable rate of loss of potential donors, with family refusal representing one of the main causes for non-effectuation of donation. In 2024, nearly half (46%) of family interviews resulted in refusal to donate organs.^3^ Other factors also contribute to the low donation rates, such as late identification of brain death and insufficient training of healthcare teams for the appropriate management of potential donors.^4^ ^,^ ^5^

Brazilian legislation on organ donation has undergone significant transformations over the past decades, reflecting the maturation of public policies and the confrontation of complex ethical dilemmas. The initial milestone was Law No. 5479/1968, which authorized the removal of organs and tissues for therapeutic purposes. In 1997, the enactment of Law No. 9434-known as the “Transplant Law”-established specific rules for organ removal and donation, adopting the presumed consent model. According to this model, all citizens would be considered donors unless they had expressed otherwise in an official document. Although this measure sought to expand the availability of organs, it generated controversies related to individual autonomy and lack of public awareness.^6^

In response to these criticisms, Law No. 10.211 came into effect in 2001, revoking presumed consent and instituting the explicit consent model, in which express family authorization is required for donation.^7^ This change represented an advance in respecting the will of donors and their families, but it also increased ethical and communicational demands, making family approach a central factor in the donation process.^6^

The regulatory framework was further consolidated with the publication of Conselho Federal de Medicina (CFM) Resolution No. 2.173/2017, in which the clinical and ethical criteria for the diagnosis of brain death were updated. This resolution promoted greater standardization and safety by more precisely defining the technical and scientific parameters required to confirm neurological death.^2^ It also granted physicians greater autonomy by allowing the withdrawal of therapeutic support after the confirmation of brain death, except in cases of potential donation.^6^

These legal milestones highlight the effort to balance respect for individual autonomy with the principles of beneficence, justice, and human dignity. At the same time, they impose growing responsibilities on healthcare professionals, requiring technical expertise, ethical sensitivity, and communication skills to conduct the donation process safely, legally, and respectfully.

The factors leading to family refusal have received increasing attention in the literature, as understanding these factors is essential for planning interventions aimed at increasing donation rates. Strategies such as strengthening the relationship between healthcare teams and families, improving communication in situations of grief, and providing training to the Intra-Hospital Commissions for Organ and Tissue Donation for Transplantation as well as the organ procurement organizations (OPOs) have proven to be fundamental in reaching this goal.^8^ ^,^ ^9^ ^,^ ^10^

Considering this context, this study aimed to evaluate the causes of brain death and the factors contributing to non-donation of organs in a quaternary hospital in São Paulo State.

## METHODS

### Study design

This coss-sectional study was conducted using data obtained from both institutional medical records and the database of the OPO of a Brazilian university hospital. The study included patients diagnosed with brain death during the study period, which enabled the evaluation of clinical, demographic, and organizational factors associated with organ donation outcomes.

### Setting

The research was based on the analysis of data collected from the medical records of patients reported with a diagnosis of brain death between 2001 and 2015, obtained from the archives of the OPO affiliated with the Hospital das Clínicas of the Faculdade de Medicina de Ribeirão Preto of the Universidade de São Paulo. Access to the data was granted through institutional authorization.

### Participants

All patients declared brain-dead during the study period were included. The selection of records was conducted on a census basis, including all available and duly registered cases in the OPO archive. No probabilistic sampling criteria were applied, thus characterizing the sample as one of convenience.

### VARIABLES

The variables analyzed included sociodemographic characteristics (sex, age), clinical factors (etiology of brain death: traumatic brain injury, hemorrhagic stroke, other neurological causes), and variables related to organ donation, such as family decision (authorization or refusal) and organs effectively donated (heart, lungs, liver, pancreas, kidneys, skin, corneas, and bones).

### Data sources and measurement

Information was extracted exclusively from the medical records archived by the OPO. Data collection was carried out manually by a nurse researcher using a structured protocol developed by the principal investigator. The instrument included both closed and open-ended items, and the data were recorded in electronic spreadsheets (Microsoft Excel^®^) to ensure standardization and systematic organization. Definitions of variables were consistently maintained throughout the collection process, minimizing measurement variability.

Potential donors were defined as patients diagnosed with brain death and eligible for organ donation according to institutional criteria. Donors were defined as patients from whom at least one organ was successfully procured. Non-donors were defined as eligible patients from whom donation did not occur.

### Bias

Because this was a retrospective study based on medical records, the main potential limitation was the incompleteness or inconsistency of medical documentation, which could introduce information bias. To mitigate this risk, only complete and legible records were included, and data collection was conducted by a trained and qualified professional.

### Quantitative variables

Continuous quantitative variables, such as age, were analyzed using measures of central tendency and dispersion. Categorical variables, such as cause of brain death or family decision, were expressed as absolute and relative frequencies.

### Statistical methods

Descriptive statistics were used to summarize the data, with categorical variables presented as frequencies and percentages, and continuous variables as mean ± standard deviation or median (interquartile range), as appropriate. Associations between categorical variables were assessed using Pearson’s chi-square test or Fisher’s exact test when applicable. To examine the relationship between the cause of brain death and organ donation decision, causes were grouped into the Neurological, Cardiac Arrest, and Other categories to ensure adequate cell counts and compliance with test assumptions.

The association between donor sex and donation actualization was evaluated through bivariate analysis using the epi.2by2() function from the epiR package, with estimation of odds ratios, relative risks, and attributable risks, along with their respective 95% confidence intervals (95% CIs). Comparisons involving continuous variables such as age were performed using Student’s t-test or the Mann-Whitney U test, depending on data distribution. All analyses were conducted using R software (R Core Team, 2023).

### Ethical approval

The study was approved by the Research Ethics Committee of the Faculdade de Medicina de Ribeirão Preto of the Universidade de São Paulo, under CAAE No. 50811615.4.3001.5440, in compliance with all ethical principles established by Resolution No. 466/2012 of the Brazilian National Health Council.

## RESULTS

**Figure 1.**
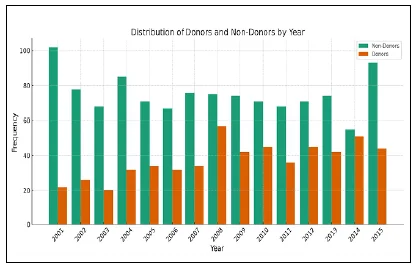
Distribution of donors and non-donors by year.

In this study, the data from 1,688 medical records, comprising 574 donors and 1,114 non-donors, were analyzed. The sociodemographic characteristics are presented in Table 1, whereas the distribution between donors and non-donors is illustrated in Figure 1. With respect to Figure 1, the year 2008 had the highest donation rate (9.8%), followed by 2009 (9.6%) and 2014 (8.5%). Regarding non-donors, we observed that the highest rate of non-donation (9.2%) was recorded in the very year that the new law (Law No. 10.211/2001) was implemented. This outcome may be related to the lack of recognition of the new law’s provisions by society, as well as insecurity arising from limited knowledge of the subject and distrust in the public health system.

**Table 1.** Sociodemographic characteristics of donors and non-donors

Sociodemographic data	Donors (n)	Donors (%)	Non-donors (n)	Non-donors (%)
Sex				
*Female*	230	40.1	443	40
*Male*	344	59.9	671	60
*Not specified*	-	-	-	-
Race/Color				
*White*	447	77.9	721	65
*Black*	64	11.1	111	10
*Mulatto*	23	4	55	5
*Brown*	34	5.9	67	6
*Asian*	1	0.2	-	-
*Not specified*	5	0.9	160	14
Age group				
*0-10 years*	22	3.8	71	6
*10-19 years*	64	11.1	89	8
*20-29 years*	76	13.2	153	14
*30-39 years*	93	16.2	201	18
*40-49 years*	134	23.3	230	21
*50-59 years*	140	24.4	216	19
≥ *60 years*	43	7.5	126	11
*Not specified*	2	0.3	28	3
Total	574	100	1,114	100

Conversely, the lowest rate of non-donations (5%) was recorded in 2014; this may be attributed to substantial federal government incentives made known through awareness campaigns and, particularly, to the enactment of Law No. 15.463 in that same year, through which “Organ Donation Month,” known as Setembro Verde (Green September), was instituted in the State of São Paulo. This most recent legislation represents a historical milestone for transplantation in Brazil, because it encourages activities for raising awareness and fosters community engagement with organ and tissue donation (Figure 1).

With regard to donors, 59.9% were male, 77.9% self-identified as White, and the majority were aged 50 to 59 years (24.4%). Similar values were identified among non-donors, of whom 60% were male and 65% were White, although the predominant age group was 40 to 49 years (21%), as shown in Table 1.

The distribution between sex and actualization of donation did not demonstrate statistical significance. The odds ratio was 0.98 (95% CI: 0.80-1.21), and the relative risk was 0.99 (95% CI: 0.92-1.07), indicating that male and female individuals had similar chances of effectuating donation. The results of the chi-square test showed no significance (χ²(1) = 0.033; p = 0.856), and those of the Fisher’s exact test confirmed the absence of association (p = 0.874). Similarly, Pearson’s chi-square analysis indicated no statistically significant association between the cause of brain death and organ donation (χ² = 4.25; df = 2; p = 0.1196).

Among non-donor patients, the main causes of brain death were neurological conditions, such as hemorrhagic stroke (33%), ischemic stroke (6%), subarachnoid hemorrhage (12%), and traumatic brain injury (26%). The most frequent reasons for non-donation of organs included cardiopulmonary arrest (40%), family refusal (32%), medical decision (11%), and positive serology (8%). Regarding the legal next of kin responsible for consent, spouses and children were the most frequent decision-makers.

## DISCUSSION

In our study, the conversion rate of patients with suspected or confirmed brain death into effective donors was 34.2%. This value is similar to that observed in a cohort of 79 patients diagnosed with brain death in Turkey, where only 32.9% became donors despite 43% of families having given consent.^11^ Furthermore, a study conducted across 10 public hospitals in the State of São Paulo reported similar results, with organ donation realized in only 236 cases (33.1%).^12^

More recent data confirm that, at approximately 46% in the past year, family adherence to organ donation after brain death remains low in Brazil.^3^ Between 2021 and 2022, approximately 45% of families refused donation, even in the presence of potential donors with confirmed diagnoses of brain death.^12^ Among the main reasons for refusing organ donation are religious factors, mistrust of the healthcare system, lack of information about the brain death and organ donation process, as well as difficulties in decision-making by family members because of bereavement.^13^

Studies indicate that the main factors contributing to non-donation include lack of knowledge about the concept of brain death, inadequate communication by healthcare teams, insufficient technical and emotional preparation of professionals for approaching families, as well as cultural and religious beliefs.^13^ ^,^ ^14^ In many cases, the absence of prior conversations about donation, coupled with the difficulty families face in accepting brain death-particularly in the presence of artificial vital signs-significantly contributes to refusal.^14^ ^,^ ^15^

Family disputes over whether to authorize or refuse donation, even when the potential donor had expressed their wishes in life, may be addressed through the Electronic Authorization for Organ Donation , a tool that remains little known and underutilized in the country. In Brazil, despite the implementation of the Electronic Authorization for Organ Donation, the process of organ donation remains legally contingent upon family consent.^16^

These findings demonstrate that, despite advances in diagnostic protocols for brain death and improvements in technical training, the realization of organ donation continues to face significant challenges. These challenges are primarily related to the subjective and emotional dimension of families, as well as structural and institutional limitations affecting the process.

### Influence of public policies and legal frameworks

The enactment of Law No. 10.211/2001 marked an important change in Brazil’s organ donation framework by establishing the requirement for explicit family consent. In this study, an increase in refusal rates was observed in 2001, which may be temporally associated with the introduction of this legal requirement and the level of public familiarity with the policy at that time. Although the requirement for family consent preserves autonomy, its implementation highlights the importance of ongoing health education and communication strategies to improve public understanding of brain death and organ donation.

In 2014, the establishment of Setembro Verde (Law No. 15.463) in São Paulo State represented a public awareness initiative aimed at promoting organ donation. In the present analysis, no significant reduction in refusal rates was observed following its implementation. This finding may be related to the limited duration of exposure within the study period. Awareness campaigns typically require sustained implementation, broad outreach, and culturally adapted strategies to influence family decision-making over time. Overall, the findings of this study suggest that isolated initiatives, even when supported by legislation, should be interpreted within the broader context of long-term public policies focused on continuous population education and professional training.

### Sociodemographic profile of donors and non-donors

The sociodemographic data revealed a predominance of male and White individuals among both donors and non-donors in this sample. This pattern may reflect the epidemiological profile of the causes of brain death-more prevalent in male individuals, and particularly from traumatic brain injury-as well as social inequalities regarding access to healthcare and information. The underrepresentation of Black and mixed-race individuals raises important questions about equity within the health system.

The predominant age group among donors (50-59 years) and non-donors (40-49 years) aligns with the epidemiology of cerebrovascular diseases and trauma, the leading causes of brain death. These findings highlight the need for primary prevention strategies in public health, especially targeting urban violence and cardiovascular risk factors.

### Causes of brain death and implications for donation

The primary causes of brain death were neurological, with traumatic brain injury a notable contributor. An analysis of 504 records in Florianópolis found that 29% of cases of brain death had neurological causes, with cerebral trauma and hemorrhagic stroke being the most prevalent.^17^ Paixão et al.,^18^ analyzing a 3-year period in a trauma and emergency hospital in Belém, found traumatic brain injury in 93% of cases. These findings support the results observed in this study, conducted in a municipality in São Paulo State.

Traumatic brain injury can lead to brain death through multiple mechanisms, including focal and diffuse injuries that cause neural disconnection and deep coma.^19^ Furthermore, increased cerebral edema may result in herniation, compromising the brainstem and inhibiting vital functions such as respiratory control. In this population, additional care-including avoidance of hypothermia, sedation, metabolic disorders, or hypotension-is required to prevent confounding factors in the diagnosis of brain death. The apnea test confirms the absence of respiratory reflexes even under strong neural drive because of carbon dioxide accumulation, which is common in individuals with neurological trauma who progress to brain death.^20^

### Determinant factors for non-donation

Over the 15-year study period, the most frequent reason for non-donation was cardiopulmonary arrest (40%), followed by family refusal (32%). In Brazil, between 2001 and 2005, the main reason for non-donation was medical contraindication, also followed by family refusal.^21^ However, between 2009 and 2015, family refusal became the most frequent cause, reaching up to 47% of cases;^21^ it remains the leading cause to this day, accounting for 46% in 2024.^12^ In the present study, however, cardiopulmonary arrest was identified as the main reason for non-donation during the period analyzed.

This discrepancy may be related to inadequate management of potential donors by healthcare teams, in addition to the prolonged interval required for clinical examinations, which-according to the former CFM resolution^22^-mandated a minimum 6-hour interval between clinical evaluations. With the current resolution,^2^ this interval was reduced to only 1 hour, which may significantly influence donation outcomes.

Regarding family refusal, several factors may contribute to its increase over time. The decision to authorize organ donation is strongly linked to trust-both in the healthcare team and in the institutions involved in the process. Lack of rapport with professionals, communication failures, and doubts regarding medical conduct at the end of life weaken acceptance of donation. Simultaneously, perceptions of injustice, corruption, and lack of transparency in the allocation system fuel concerns about whether the process is ethically conducted. Institutional insecurity and interpersonal mistrust constitute additional barriers that must be addressed through qualified communication strategies, process audits, and greater transparency.^13^ ^,^ ^23^

Healthcare teams play a fundamental role in the identification and management of potential organ donors. Professionals must recognize patients with suspected brain death at an early stage, perform diagnostic tests in accordance with legislation, and manage hemodynamic changes until the protocol is completed. At the same time, it is essential to provide support and clear information to families throughout the process. However, many professionals report difficulties in performing this task, attributed to several factors, such as gaps in academic training, lack of specific education on diagnosing brain death, insufficient preparation for family discussions about organ donation, and insecurity in managing physiological changes during the process.^24^

The lack of professional training in donor management, combined with family insecurity and the absence of rapport with healthcare teams, may compromise the donation process, leading to increased family refusals or loss of potential donors because of clinical conditions that could otherwise be adequately managed.

### Limitations

This study had limitations inherent to an observational and retrospective design, which allowed only the identification of associations without establishment of causality, making it subject to uncontrolled biases and residual confounding from unmeasured variables. Moreover, its cross-sectional nature precluded the analysis of temporal changes, and the generalizability of the findings should be interpreted with caution, as they reflect the specific reality of a single-center study.

## CONCLUSION

In this study, we sought to evaluate the causes of brain death and the factors associated with non-donation of organs. The findings suggest that although neurological causes predominate in brain death, the realization of donation is more strongly conditioned by social and organizational aspects-such as family refusal and trust in the healthcare system-than by demographic and clinical characteristics. The temporal variation observed indicates that public policies and awareness campaigns, such as Setembro Verde, may play a decisive role in raising societal awareness.

Thus, expanding public access to information, strengthening institutional transparency, and fostering dialogue with families are central strategies to overcoming barriers to donation. Prospective and interventional studies are needed to deepen the understanding of these determinants and to test approaches capable of increasing the availability of organs for transplantation.

## Data Availability

Data supporting the findings of this study are available upon request from the corresponding author, Vagner Pires de Campos Junior.
